# Strong Association of the rs4986790 Single Nucleotide Polymorphism (SNP) of the Toll-Like Receptor 4 (*TLR4*) Gene with Human Immunodeficiency Virus (HIV) Infection: A Meta-Analysis

**DOI:** 10.3390/genes12010036

**Published:** 2020-12-30

**Authors:** Yong-Chan Kim, Byung-Hoon Jeong

**Affiliations:** 1 Korea Zoonosis Research Institute, Jeonbuk National University, Iksan, Jeonbuk 54531, Korea; kych@jbnu.ac.kr; 2 Department of Bioactive Material Sciences and Institute for Molecular Biology and Genetics, Jeonbuk National University, Jeonju, Jeonbuk 54896, Korea

**Keywords:** HIV, susceptibility, TLR4, rs4986790, SNP, D299G, meta-analysis

## Abstract

Human immunodeficiency virus (HIV) causes acquired immune deficiency syndrome (AIDS) and enters the host cell via CD4 and either CC-chemokine receptor 5 (CCR) or CXC-chemokine receptor 4 (CXCR4). HIV is directly recognized by toll-like receptor 4 (TLR4) and affects downstream immune-related signal pathways. In addition, stimulated TLR4 inhibits HIV-1 invasion, and the rs4986790 single nucleotide polymorphism (SNP) (D299G) of the *TLR4* gene contributes to the risk of HIV-1 infection in an Indian population. To evaluate whether the rs4986790 SNP of the *TLR4* gene is related to vulnerability to HIV-1 infection, we collected genetic information from HIV-1 patients in previous studies and performed an association analysis with a matched control population obtained from the 1000 Genomes Project. In addition, to strengthen the results of association analysis, we performed a meta-analysis. We identified a strong association between the rs4986791 SNP and susceptibility to HIV infection in HIV-infected patients in previous studies and a matched control population obtained from the 1000 Genomes Project. In addition, we found that the G allele of the rs4986791 SNP in the *TLR4* gene is strongly related to susceptibility to HIV infection in three Caucasian populations (odd ratio = 2.29, 95% confidence interval: 1.72–3.07, *p* = 1.438 × 10^−7^) and all four populations (odd ratio = 2.22, 95% confidence interval: 1.74–2.84, *p* = 2 × 10^−10^) in a meta-analysis. To the best our knowledge, this was the first meta-analysis on the association between the rs4986791 SNP of the *TLR4* gene and susceptibility to HIV infection.

## 1. Introduction

Human immunodeficiency virus (HIV) is a retrovirus that harbors positive sense single strand RNA as its viral genome. The viral genome is translated to viral structural proteins (Gag, Env and Pol), essential regulatory elements (Tat and Rev) and accessory regulatory proteins (Nef, Vpr, Vif and Vpu) [1,2]. It has been postulated that HIV originated from nonhuman primates and spread to humans through certain body fluids, including blood, semen, vaginal or rectal fluids and breast milk during the 1900s. HIV is internalized by host cells using host receptor proteins, including CD4 and either CC-chemokine receptor 5 (CCR) or CXC-chemokine receptor 4 (CXCR4). Thus, HIV mainly targets CD4^+^ T cells and can lead acquired immune deficiency syndrome (AIDS) by disarming the host immune system [3,4].

To prevent external infection, several pattern recognition receptors (PRRs) including toll-like receptors (TLRs), RIG-I-like receptors (RLRs) and NOD-like receptors (NLRs), recognize a pathogen-related pattern of external invaders and activate the host immune system through downstream regulators, including nuclear factor kappa-light-chain-enhancer of activated B cells (NF-κB), mitogen-activated protein kinase (MAPK) and interferon type I [5,6,7,8,9]. Among TLRs, a previous study reported that TLR4 located on the cell surface was upregulated in response to HIV-1 infection in monocyte-derived macrophage (MDM) and peripheral blood mononuclear cell (PBMC) [10]. In addition, the Tat protein of HIV-1 directly binds to TLR4 and activates tumor necrosis factor-α (TNF-α) and interleukin-10 (IL-10) [11]. These studies suggest an association between TLR4 and HIV infection. Remarkably, TLR4 stimulation protects HIV infection from CD4^+^ T cells in vitro [12]. In addition, a functional variation of TLR4, namely, the rs4986790 single nucleotide polymorphism (SNP) (D299G), contributes to risk of HIV-1 infection in an Indian population [13].

To validate whether the rs4986790 SNP of the *TLR4* gene is associated with susceptibility to HIV-1 infection, we collected three studies that contain genetic information on ethnic backgrounds and allele frequencies of rs4986790 SNP of the *TLR4* gene from HIV-infected patients [14,15,16]. The matched Caucasian control populations, including Iberian populations from Spain, Tuscans from Italy and northern and western Europeans from Utah, were obtained from the 1000 Genomes Project and used for an association analysis [17]. Then, we performed a meta-analysis by collecting data from eligible studies to evaluate the association between rs4986790 SNP of the *TLR4* gene and susceptibility to HIV-1 infection.

## 2. Materials and Methods

### 2.1. Literature Search

A literature search was conducted in PubMed to identify studies reporting the rs4986790 SNP of the *TLR4* gene. The following searching terms were used: “TLR4,” “SNP” and “HIV” combined with “polymorphism” or “susceptibility” (the last search update was performed on 18 July 2020). Irrelevant reports were excluded after the initial screening of titles and abstracts. Eligible studies should comply with the following inclusion criteria: (1) investigating the association between rs4986790 and HIV-1; (2) a cohort or case–control study; (3) genetic information of rs4986790 of HIV-1 infected patients (4) with full text; (5) published in English. Exclusion criteria were as follows: (1) animal studies; (2) case reports or reviews; (3) containing insufficient genotype data.

### 2.2. Association Analysis

We collected three studies that contain information on ethnic background and allele frequencies of the rs4986790 SNP of the *TLR4* gene in HIV-infected patients [12,13,14]. Matched Caucasian control populations, including Iberian populations from Spain, Tuscans from Italy and northern and western Europeans from Utah, were obtained from the 1000 Genome Project [17]. Differences in allele frequencies between HIV-infected patients and control populations were analyzed using SAS version 9.4 (SAS Institute Inc., Cary, NC, USA). Statistical significance was determined by *p*-values obtained using the χ^2^ test.

### 2.3. Meta-Analysis

The strength of the association between the rs4986790 SNP of the *TLR4* gene and susceptibility to HIV infection was evaluated by calculating crude additive odd ratios and 95% confident intervals for each study. The pooled odd ratios were calculated based on an additive genetic model (A allele vs. G allele). Heterogeneity was based on *p*-value and I^2^ value. A fixed effect model was used to calculate the pooled odd ratios. Publication bias was examined using Begg’s funnel plot and Egger’s weighted regression methods. All statistical analyses were conducted using the meta package of R program (https://www.r-project.org/).

## 3. Results

### 3.1. Strong Association between the rs4986790 SNP (D299G) of the TLR4 Gene and Susceptibility to HIV Infection in Three Caucasian Populations

We searched 20 research articles following searching terms: “TLR4,” “SNP” and “HIV” combined with “polymorphism” or “susceptibility” (the last search update was performed on 18 July 2020) on PubMed. After excluding duplicate articles, a total of four relevant studies were extracted from the databases based on our inclusion and exclusion criteria.

To identify an association between the rs4986790 SNP (D299G) and susceptibility to HIV infection, we performed an association analysis between HIV patients for whom information on ethnic background and allele frequencies of rs4986790 SNP of the *TLR4* gene were reported in previous studies [14,15,16] and matched Caucasian control populations, including Iberian populations in Spain, Tuscans from Italy and northern and western Europeans from Utah, obtained from the 1000 Genomes Project. Notably, allele frequencies of the rs4986790 SNP of the *TLR4* gene exhibited a strong association (*p* < 0.05) with susceptibility to HIV infection in all tested groups (Table 1).

### 3.2. Strong Association between the rs4986790 SNP of the TLR4 Gene and Susceptibility to HIV Infection Based on a Meta-Analysis

**Figure 1:**
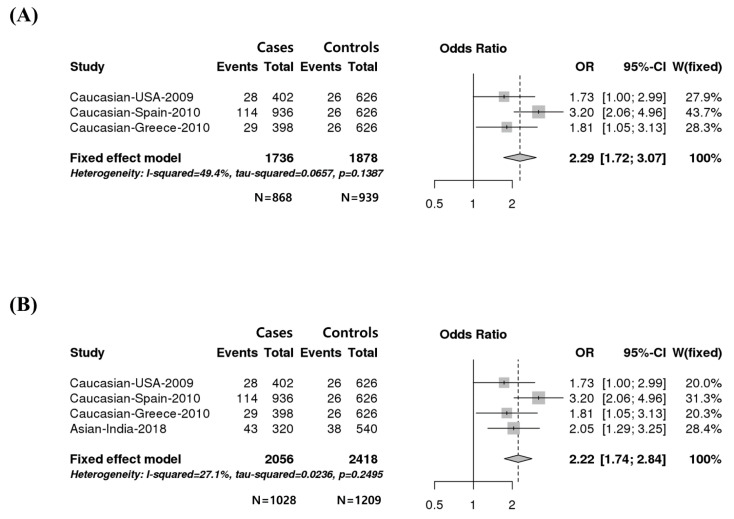
(**A**) Forest plot of the association between the rs4986790 single nucleotide polymorphism (SNP) of the *TLR4* gene and susceptibility to human immunodeficiency virus (HIV) infection in a Caucasian population. (**B**) Forest plot of the association between the rs4986790 SNP and susceptibility to HIV infection in four populations. Forest plots of odd ratios (ORs) were calculated using an additive model (A allele vs. G allele). The number of each column of “Cases” and “Controls” indicates number of alleles. “N” indicates the number of people.

A total of four studies that reported the association between the rs4986790 SNP and susceptibility to HIV infection were identified from the literature and included in this meta-analysis (Figure 1). In total, 1028 HIV-infected patients and 1209 controls were included in the meta-analysis (Figure 1B). Detailed information on the eligible studies is presented in Table 1.

Heterogeneity among collected studies was tested using the *p*-value and I^2^ value (Table 2). Given that heterogeneity was not found in these studies, we used a fixed effect model for the meta-analysis. In addition, given that the genetic information from previous studies was commonly available as allele frequencies, we used an additive model (A allele vs. G allele) for a meta-analysis. Our data revealed an association with the risk of HIV infection in Caucasian populations (odd ratio = 2.29, 95% confidence interval: 1.72–3.07, *p* = 1.438 × 10^−7^) and all four populations (odd ratio = 2.22, 95% confidence interval: 1.74–2.84, *p* = 2 × 10^−10^) (Figure 1).

**Figure 2:**
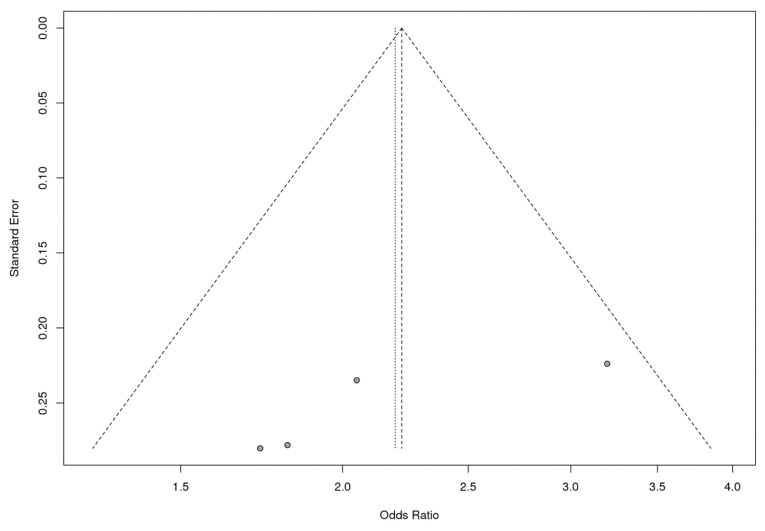
Begg’s funnel plot for the meta-analysis of the rs4986790 SNP of the *TLR4* gene in four populations.

To examine potential publication bias, Begg’s and Egger’s tests were performed. The shape of the funnel plots revealed no evidence of obvious asymmetry (Figure 2). However, Egger’s test indicated the possibility of publication bias in a meta-analysis of the Caucasian subgroup (*p* = 0.0208) (Table 2). Sensitivity analyses were conducted to evaluate the impact of single studies on the pooled results by omitting individual studies in turn. In the Caucasian subgroup and the total group, after excluding one study (Caucasian-Spain-2010), the remaining studies showed similar results (the relative results are not provided in the text).

## 4. Discussion

TLR4 is located on the cell surface and senses bacterial lipopolysaccharide (LPS). Thus, previous studies have revealed the association between TLR4 and bacterial-related phenotypes, including tuberculosis, cirrhosis, ascites, scrub typhus and Crohn’s disease [18,19]. In this context, the rs4986790 SNP (D299G) of the *TLR4* gene has also been investigated to explain the variation of disease-related phenotypes based on the risk allele of the rs4986790 SNP. In addition, differences in the local crystal structure of the TLR4-MD2-LPS complex were noted between TLR4 with the wild type allele and the G299 allele [20,21]. Since genetic variations confer functional alteration, these studies indicate that the rs4986790 SNP of the *TLR4* gene is related to anti-bacterial functions of TLR4 [22,23,24]. Interestingly, TLR4/MD2 complex directly interacts with the Tat protein of HIV-1, and HIV-1 modulates the expression level of TLR4 and downstream immune responses, including the NF-κB pathway [25,26]. In addition, the rs4986790 SNP is associated with the risk of HIV-1 infection in an Indian population [13]. Since TLR4 plays a dual role in HIV-1 infection and anti-bacterial function, rs4986790 SNP, which is associated with structural and functional alteration of TLR4, showed a relationship with the susceptibility of HIV infection and bacterial-related phenotype, including tuberculosis. These studies indicate the possibility of the association between the rs4986790 SNP of the *TLR4* gene and susceptibility to HIV infection. Thus, we performed an association analysis in Caucasian HIV-infected patients from previous studies. When considering the ethnic background of Caucasian HIV-infected patients, genetic information on control populations was not available in the previous Caucasian studies. Thus, we selected and matched control populations, including Iberian populations from Spain, Tuscans from Italy and northern and western Europeans from Utah from the 1000 Genomes Project. Remarkably, we identified a strong association between the rs4986790 SNP and susceptibility to HIV infection in all tested groups (Table 1). However, since these results were highly dependent on the frequency of the control Caucasian population from the 1000 Genome Project, further validation is highly desirable in other control Caucasian populations. Furthermore, a case-control study using direct sequencing from HIV-1 infected patients in Caucasians, North and South Americans and East Asians has not been performed thus far. Thus, further study to confirm an association between rs4986790 SNP and vulnerability of HIV-1 infection is needed. The number of each column of “Allele frequencies” indicates number of alleles. “Total, n” indicates the number of people. Bold text indicates statistically significant results (*p* < 0.05). The control population obtained from 1000 Genome Project contains Iberian populations from Spain (IBS), Tuscans from Italy (TSI) and northern and western Europeans from Utah (CEU). To strengthen the conclusions, we performed a meta-analysis. Notably, we identified strong associations in Caucasian and all four populations (Figure 1, Table 2). Although publication bias was found in the Caucasian population, both heterogeneity and publication biases were alleviated by omitting one study (Caucasian-Spain-2010). Given that association studies on the *TLR4* gene in HIV-infected patients are rare, further investigations using a larger number and various ethnic groups, including Africans and East Asians, are highly desirable in the future. In addition, given that another SNP, namely, the rs4986791 SNP (T399I) of the *TLR4* gene, is also associated with bacterial infection, further validation of the association of rs4986791 SNP in HIV-infected patients should be performed in future studies.

## 5. Conclusions

In conclusion, we identified a strong association between the rs4986791 SNP and susceptibility to HIV infection between HIV-infected patients in previous studies and a matched control population obtained from the 1000 Genomes Project. We also found that the G allele of the rs4986791 SNP of the *TLR4* gene is potent risk factors of HIV infection in Caucasians and all four populations assessed in this meta-analysis. To the best of our knowledge, this was the first meta-analysis assessment of the association between the rs4986791 SNP of the *TLR4* gene and susceptibility to HIV infection.

## Figures and Tables

**Table 1:** Comparison of allele frequencies of rs4986790 (D299G) in the toll-like receptor 4 (*TLR4*) gene between human immunodeficiency virus (HIV)-infected patients in previous studies and matched control populations obtained from the 1000 Genomes Project.

Population	Country	Year of Publication	Authors	Cases			Controls			*p*-Value	Reference
				Allele Frequencies, n (%)		Total, n	Allele Frequencies, n (%)		Total, n		
				A	G		A	G			
Caucasian	USA	2009	Pine	374 (93.03)	28 (6.97)	201	600 (95.85)	26 (4.15)	313	0.0486	1000 Genomes Project, [14]
Caucasian	Spain	2010	Pulido	822 (87.82)	114 (12.18)	468	600 (95.85)	26 (4.15)	313	5.2687 × 10^−8^	1000 Genomes Project, [15]
Caucasian	Greece	2010	Papadopoulos	369 (92.71)	29 (7.29)	199	600 (95.85)	26 (4.15)	313	0.0302	1000 Genomes Project, [16]
Asian	India	2018	Vidyant	277 (86.56)	43 (13.44)	160	502 (92.96)	38 (7.04)	270	0.0019	[13]

**Table 2:** Meta-analysis of the association between the rs4986790 SNP of the *TLR4* gene and susceptibility to HIV infection.

Populations	Adjust *p*-Value	Heterogeneity		Egger’s Test
		*p*-Value	I^2^ Value	
Caucasian	1.438 × 10^−7^	0.1387	49.4%	0.0208
Total	2 × 10^−10^	0.2495	27.1%	0.1598

## Data Availability

All data generated or analysed during this study are available from the corresponding author on reasonable request.

## Abbreviations

HIV	Human immunodeficiency virus
SNP	Single nucleotide polymorphism
TLR4	Toll-like receptor 4
AIDS	Acquired immune deficiency syndrome
CCR	CC-chemokine receptor 5
CXCR4	CXC-chemokine receptor 4
NF-κB	Nuclear factor kappa-light-chain-enhancer of activated B cells
TNF-α	Tumor necrosis factor-α
